# Comparison of Clinical Laboratory Standards Institute (CLSI) Microdilution Method and VITEK 2 Automated Antifungal Susceptibility System for the Determination of Antifungal Susceptibility of Candida Species

**DOI:** 10.7759/cureus.20220

**Published:** 2021-12-06

**Authors:** Burcu Dalyan Cilo, Beyza Ener

**Affiliations:** 1 Clinical Microbiology, University of Health Sciences, Bursa Yuksek Ihtisas Training & Research Hospital, Bursa, TUR; 2 Microbiology, Microbiology, Uludag University Medical Faculty, Bursa, TUR

**Keywords:** candida infections, minimum inhibitory concentration, antifungal susceptibility, candida species, microbroth dilution

## Abstract

Introduction

Changes in the epidemiology of *Candida* infections, increasing resistance, and advances in treatment have increased the need to perform antifungal susceptibility testing in clinical laboratories. Standardized reference, the microbroth dilution method, and various commercial antifungal susceptibility test systems are used to determine antifungal susceptibility. This study aims to determine and compare the antifungal susceptibility of various *Candida *species isolated from blood cultures in our laboratory with the CLSI M27 microdilution reference method and VITEK 2 automated system (bioMérieux, Marcy-l'Étoile, France).

Methods

The antifungal susceptibility of a total of 140 *Candida* strains to fluconazole, voriconazole, and amphotericin B, and a total of 92 strains to anidulafungin was tested with the CLSI M27 method and the VITEK 2 automated system. For fluconazole, voriconazole, and amphotericin B, essential and categorical agreement percentages were calculated between the two methods. Because there is no anidulafungin in the VITEK 2 system, anidulafungin results obtained with CLSI were compared with micafungin only in terms of categorical agreement. In the category comparison, CLSI clinical breakpoints were used; the epidemiological cut-off values were used when they were not available. Very major error, major error, and minor error rates were calculated.

Results

In general, the minimum inhibitory concentration (MIC) values obtained with VITEK 2 for azole group drugs were found to be one-fold higher than the CLSI MICs read at the 24th hour. While the essential agreement between the two methods was >90% for amphotericin B and voriconazole, it remained at 85% for fluconazole. Overall, the best categorical agreement was obtained with amphotericin B (99.3%), and the least categorical agreement was obtained with voriconazole (85.7%). A very major error was seen with amphotericin B (0.7%) and fluconazole (0.7%) in one *C. parapsilo*sis strain each. No resistance was detected with VITEK 2 in one *C. glabrata* strain found to be resistant to fluconazole by the reference method. Major and minor error rates were higher for azole drugs than amphotericin B and anidulafungin/micafungin.

Conclusion

The VITEK 2 system is a fast and highly applicable system, and with these features, it is advantageous for routine laboratories. In this study, although the error rate was not very high, one fluconazole-resistant *C. parapsilosis* and *C. glabrata* strain could not be detected with VITEK 2. The increase in data on the antifungal performance of the VITEK 2 system, which is available in many routine laboratories due to its ability to be used for bacteria identification and sensitivity, will contribute to the usability of the system for this purpose. In this study, data that will support the literature information in terms of the antifungal performance of the VITEK 2 system are presented.

## Introduction

Infections caused by fungi have become more prevalent due to advances in diagnosis and treatment. The increase in the number of immunocompromised patients, the prominence of major surgical operations, more frequent use of interventional procedures such as the use of catheters, and the widespread use of antibiotics pose a risk for opportunistic fungal infections [1]. *Candida* *(C.)* species are the most common cause of opportunistic mycoses and are among the leading nosocomial infections [2]. Although the causative agent responsible for most of the infections is *C. albicans*, the increase in infections caused by other *Candida* species is noteworthy [3]. In invasive* Candida* infections, early diagnosis and treatment are important to reduce mortality and morbidity [4]. In vitro antifungal susceptibility tests are important in choosing the most appropriate agent to be used in treatment. In recent years, due to the change in epidemiology and the increase in the isolation of potentially resistant strains, the development of new antifungal drugs to be used in the treatment, the increase in antifungal resistance, and the development of susceptibility test methods, antifungal susceptibility tests have been applied more frequently in clinical laboratories [5].

 The reference method for antifungal susceptibility tests is the microbroth dilution method standardized by the “Clinical and Laboratory Standards Institute” (CLSI) and “European Committee for Antimicrobial Susceptibility Testing” (EUCAST) [6-7]. It is a laborious and time-consuming method due to manual plate preparation and its results are difficult to interpret, and this requires experience, and so this method is difficult to apply in routine laboratories. With the standardization of antifungal susceptibility methods, various automatic or semi-automatic commercial antifungal susceptibility testing systems that are easy to apply in clinical laboratories have been developed [8-9]. Today, the disk diffusion method standardized by CLSI, as well as the gradient diffusion-based Etest (bioMérieux, Marcy-l'Étoile, France), the commercial Sensititer YeastOne system based on the colorimetric microbroth dilution principle (TrekDiagnosticSystems Ltd., East Grinstead, England), and the commercial full automated VITEK 2 (bioMérieux) systems are used to determine antifungal susceptibility [10-11].

The VITEK 2 system (bioMérieux) is a fully automated commercial system that evaluates yeast growth spectrophotometrically and is capable of working for fungal identification and antifungal susceptibility simultaneously. The biochemical characteristics of the agent are determined with VITEK 2 YST cards, and species are identified by comparing with a large database [12-15]. The susceptibility of different antifungals can be tested with AST antifungal susceptibility cards (YS01, YS02, YS06, YS07, YS08, YS09) [16].

AST cards are essentially a miniaturized version of the two-fold dilution method used to determine the minimum inhibitory concentration (MIC) in µg/ml by the microdilution method. It consists of 64 wells containing aliquots of a specific antifungal agent. After the card is placed in the device with the appropriate microorganism suspension, no further action is required. By vacuuming, the suspension is drawn to the card, then sealed and automatically placed in the reader/incubator. The system includes a software program that validates and interprets susceptibility test results. Microorganism growth is evaluated based on the attenuation of the light measured by the optical scanner, and these data are used to determine MIC values ​​for antifungal agents. MIC values ​​can be determined after 9.1 to 27.1 hours of incubation (mean 12 to 14 hours) [16].

This study aimed to determine and compare the antifungal susceptibility of various *Candida* species isolated from blood cultures in our laboratory with the CLSI reference method and VITEK 2 automated system using the AST-YS08 cards and to investigate the applicability of the automated antifungal susceptibility system in clinical laboratories.

## Materials and methods

In this study,* Candida* strains isolated from blood cultures and identified by germ-tube test, morphology in cornmeal-tween 80 medium, and API ID32 C (bioMérieux) system were used. The isolates stored at -80°C in the stocks were revived by passage twice in Sabouraud Dextrose Agar (SDA) and pure culture was obtained. Quality control was achieved with *C. parapsilosis* ATCC 22019 and *C.krusei* ATCC 6258 strains.

In-vitro susceptibility of 140 strains identified as *C. albicans* (n=46), *C. parapsilosis* sensu stricto (n=41) [17], *C. glabrata* species complex (n=35),* C. tropicalis* (n=18) against fluconazole (Sigma Aldrich, St. Louis, MO), voriconazole (Pfizer Central Research, New York, NY), and amphotericin B (Amresco, OH) were determined by the broth microdilution method recommended in the CLSI M27 guideline [6]. Anidulafungin susceptibility was measured in 92 isolates (*C. albicans*=23,* C. glabrata* species complex=28, *C. parapsilosis* sensu stricto=30, *C. tropicalis*=11). Each strain belonged to a different patient.

According to CLSI recommendations, antifungal drugs dissolved in dimethylsulfoxide (DMSO) were diluted in RPMI 1640 medium containing 0.2% glucose and distributed at the appropriate concentration on U-bottom microdilution plates. The inoculum suspension was adjusted to a final concentration of 0.5x103-2.5x103 cells/ml and was dispensed into microdilution wells with different antifungal concentrations. Plates were incubated at 35°C and were visually evaluated after 24 hours. In cases where growth was insufficient, the incubation was extended to 48 hours. For amphotericin B, the well in which growth was completely inhibited compared to the control well, and for fluconazole, voriconazole, and anidulafungin, the well where growth was significantly reduced was determined as MIC [6].

In the determination of antifungal susceptibility with the automated system, the turbidity of *Candida *strains was adjusted to 2.0 McFarland (1.8-2.2; DensiCheck, BioMérieux) with 0.45% sterile NaCl in accordance with the manufacturer's recommendations. They were loaded to the VITEK 2 AST YS08 fungal susceptibility card (BioMérieux) and the cards were placed into the instrument. This card measures the susceptibility against amphotericin B (≤0.25->16 µg/ml), flucytosine (≤1->64µg/ml), fluconazole (≤0.5->64µg/ml), voriconazole (≤0.125->8µg/ml), caspofungin (≤0,125->8 µg/ml), and micafungin (≤0.06->8µg/ml) [16]. However, in this study, flucytosine and caspofungin were excluded from the evaluation because flucytosine was not available in our country and the latter produces variable results in in-vitro susceptibility. Since anidulafungin is not included in the VITEK 2 AST-YS cards, the anidulafungin results obtained with the CLSI were compared with micafungin only for categorical agreement (CA).

Except for the anidulafungin/micafungin results, if the MIC values obtained by both methods were within ± 2 dilution limits, they were considered compatible and the agreement rates were calculated as percentages. Clinical breakpoints (CBs) and Epidemiological Cut-Off values (ECOFFs) in the CLSI guidelines were used to determine the CA [18-19]. While the fluconazole CBs for* C. albicans, C. parapsilosis,* and *C. tropicalis* were taken as “≤2 µg/mL (susceptible:S); 4 µg/mL (susceptible dose-dependent: SDD); ≥8 µg/mL (resistant: R)”, for *C. glabrata*, they were taken as “≤32 µg/mL (SDD) and≥64 µg/mL (R)”. While the CBs for voriconazole for *C. albicans, C. parapsilosis,* and *C. tropicalis* were taken as “≤0.12µg/mL (S), 0.25-0.5 µg/mL (SDD), and ≥1 µg/mL (R)", ECOFFs were used as there were no clinical breakpoints in *C. glabrata*. Strains with voriconazole ECOFFs (0.25 µg/mL) and below were considered as “wild-type (WT),” and strains above it were considered as non-WT (NWT) [19]. The CBs of anidulafungin for *C. albicans* and *C. tropicalis* are taken as “≤0.25 µg/mL (S), as 0.5 µg/mL (intermediate =I), ≥1 µg/mL (R)”; while for *C. glabrata,* they were taken as “≤0.125 µg/mL (S), 0.25 µg/mL (I), ≥0.5 µg/mL (R)”, and for *C. parapsilosis* as “≤2 µg/mL (S),4 µg/mL (I), ≥8 /mL (R)” [18]. Since there were no CBs for amphotericin B, ECOFFs were used. The ECOFFs for amphotericin B were taken as 2 µg/mL for *C. albicans, C. glabrata,* and *C. tropicalis;* it was taken as 1 µg/mL for *C. parapsilosis* and the strains above it were evaluated as NWT [19]. MIC values determined by the reference microbroth dilution method and VITEK were accepted as findings of CA when they were determined as S/WT or R/NWT. The MIC determined by the reference microbroth dilution method was R/NWT, MIC determined by VITEK 2 as S/WT was regarded as a very major error while MIC determined by the reference microbroth dilution method, as S/WT, MIC determined by VITEK 2 as R/NWT was considered a major error. It was accepted as a minor error if the strain was found susceptible or resistant by one method and SDD/I by the other method.

## Results

In Table 1, the MIC ranges, MIC_50_, MIC_90 _values ​​of the species determined by both methods against antifungal drugs, and the essential agreements (EA) between the two methods are given. While the total amphotericin B MIC_90_ value was found to be the same as 1 µgr/ml with both methods, fluconazole and voriconazole MIC_90_ values ​​were found to be one-fold higher in the VITEK 2 method. As seen in the table, the EA between the two methods in total was >90% for amphotericin B and voriconazole while it remained at 85% for fluconazole. When the species were evaluated individually, the worst agreement (75.6%) was obtained for fluconazole in *C. parapsilosis*.

**Table 1:** The susceptibilities determined by the reference CLSI and VITEK 2 methods and the category agreement between the two methods VME: very major error, ME: major error, MIE: minor error, CLSI: Clinical Laboratory Standards Institute

Species	Antifungal Drug	Method	Number of Isolates by category (%)			Category agreement (%)	VME (%)	ME (%)	MIE (%)
			S/WT	SDD/I	R/ nonWT				
C. albicans	Amphotericin B (46)	CLSI	46 (100)			100			
		VITEK	46 (100)						
	Fluconazole (46)	CLSI	46 (100)			95.6		1 (2.2)	1 (2.2)
		VITEK	44 (95.6)	1 (2.2)	1(2.2)				
	Voriconazole (46)	CLSI	46 (100)			91.3		4 (8.7)	
		VITEK	42 (91.3)		4 (8.7)				
	Anidula/Mica (23)	CLSI	23 (100)			100			
		VİTEK	23 (100)						
C. parapsilosis	Amphotericin B (41)	CLSI	40 (97.6)		1 (2.4)	97.6	1 (2.4)		
		VITEK	41 (100)						
	Fluconazole (41)	CLSI	29 (70.7)	1 (2.4)	11 (26.8)	82.9	1 (2.4)	3 (7.3)	3 (7.3)
		VITEK	26 (63.4)	2 (4.9)	13 (31.7)				
	Voriconazole (41)	CLSI	35 (85.4)	3 (7.3)	3 (7.3)	75.6		2 (4.8)	8 (19.5)
		VITEK	27 (65.8)	9 (21.9)	5 (12.2)				
	Anidula/Mica (30)	CLSI	29 (96.7)	1 (3.3)		80			6 (20)
		VİTEK	25 (83.3)	5 (16.7)					
C. glabrata	Amphotericin B (35)	CLSI	35 (100)			100			
		VITEK	35 (100)						
	Fluconazole (35)	CLSI		34 (97.1)	1 (2.9)	97.1			1 (2.9)
		VITEK		35 (100)					
	Voriconazole (35)	CLSI	34 (97.1)		1 (2.9)	82.8		2 (5.7)	
		VITEK	32 (91.4)		3 (8.6)				
	Anidula/Mica (28)	CLSI	28 (100)			100			
		VITEK*	28 (100)						
C. tropicalis	Amphotericin B (18)	CLSI	18 (100)			100			
		VITEK	18 (100)						
	Fluconazole (18)	CLSI	18 (100)			88.9		2 (11.1)	
		VITEK	16 (88.9)		2 (11.1)				
	Voriconazole (18)	CLSI	16 (88.9)	2 (11.1)		77.8		1 (5.5)	3 (16.7)
		VITEK	16 (88.9)	1 (5.5)	1 (5.5)				
	Anidula/Mica (11)	CLSI	10 (90.9)	1 (9.1)		90.9		1 (9.1)	
		VITEK	9 (81.8)	1 (9.1)	1 (9.1)				
Total	Amphotericin B (140)	CLSI	139 (99,3)		1 (0.7)	99,3	1 (0.7)		
		VİTEK	140 (100)						
	Fluconazole (140)	CLSI	93 (66.4)	35 (25)	12 (8.6)	91.4	1 (0.7)	6 (4.3)	5 (3.6)
		VİTEK	86 (61.4)	38 (27.1)	16 (11.4)				
	Voriconazole (140)	CLSI	127 (90.7)	5 (3.6)	8 (5.7)	85.7		9 (6.4)	11 (7.9)
		VİTEK	117 (83.6)	10 (7.1)	13 (9.3)				
	Anidula/Mica (92)	CLSI	90 (97.8)	2 (2.2)		92.4		1 (1.1)	
		VİTEK	85 (92.4)	6 (6.5)	1 (1.1)				6 (6.5)

Between the two methods, the best CA was obtained with amphotericin B (99.3%), and the worst CA was obtained with voriconazole (85.7%). Amphotericin B was found to be a 100% effective drug with both methods in almost all of the strains. Only one *C. parapsilosis* strain was found to be NWT by the CLSI method while WT was found by VITEK. After amphotericin B, anidulafungin/micafungin was found to be the best drug in terms of both efficacy and compliance. In azoles, the agreement between the two methods was relatively low (Table 2).

**Table 2:** Antifungal susceptibility results determined by the reference CLSI and VITEK 2 methods and the essential agreement between the two methods EA: essential agreement, CLSI: Clinical Laboratory Standards Institute

Species	Antifungal Drug	Method	MIC Range	MIC_50_	MIC_90_	EA (%)	
								Amphotericin B	CLSI	≤0.0313- 1	0.5	1	93.4	
		VITEK	≤0.25-1	0.5	1		
C. albicans (46)	Fluconazole	CLSI	≤0.125- 2	0.25	0.25	89.1	
		VITEK	≤0.5- 8	1	1		
	Voriconazole	CLSI	≤0.0313- 0.0625	0.0313	0.0625	91.3	
		VITEK	≤0.125- 1	≤0.125	≤0.125		
	Amphotericin B	CLSI	0.0625-4	0.5	2	90.2	
		VITEK	≤0.25-1	0.5	1		
C. parapsilosis (41)	Fluconazole	CLSI	≤0.125- >64	0.5	8	75.6	
		VITEK	≤0.5->64	2	>64		
	Voriconazole	CLSI	≤0.0313- 1	0.0625	0.25	90.2	
		VITEK	≤0.125- 2	≤0.125	0.5		
	Amphotericin B	CLSI	0.125- 1	1	1	100	
		VITEK	≤0.25- 1	0.5	1		
C. glabrata (35)	Fluconazole	CLSI	≤0.125-64	1	16	88.6	
		VITEK	2- 32	2	16		
	Voriconazole	CLSI	≤0.0313- 2	0.0625	0.5	91.4	
		VITEK	≤0.125- 4	≤0.125	0.25		
	Amphotericin B	CLSI	0.25- 1	1	1	100	
		VITEK	≤0.25- 1	0.5	0.5		
C. tropicalis (18)	Fluconazole	CLSI	≤0.125- 2	0.5	1	88.9	
		VITEK	≤0.5- >64	1	2		
	Voriconazole	CLSI	≤0.0313- 0.25	0.0625	0.125	88.9	
		VITEK	≤0.125- 2	≤0.125	≤0.125		
	Amphotericin B	CLSI	≤0.0313- 4	1	1	93.5	
		VITEK	≤0.25-1	0.5	1		
TOTAL (140)	Fluconazole	CLSI	≤0.125 - >64	0.5	8	85.0	
		VITEK	≤0.5->64	1	16		
	Voriconazole	CLSI	≤0.0313- 2	0.0625	0.25	90.0	
		VITEK	≤0.125- 4	≤0.125	0.5		

All *C. albicans* strains tested with the CLSI reference method were S/WT against four antifungals. However, strains with reduced susceptibility to azoles (voriconazole and fluconazole) were found with VITEK 2. Although there was no very major error between the two methods, major and minor errors were found due to the high MIC values ​​obtained with VITEK 2 in azoles (Table 1).

*C. parapsilosis* strains were less sensitive to fluconazole (70.7%) and voriconazole (63.4%) by the reference method. Since the MIC values ​​obtained with fluconazole, voriconazole, and micafungin were higher with VITEK 2, major and minor errors were encountered and one strain resistant to fluconazole was found to be susceptible to VITEK 2 and there was a major error (Table 2).

A fluconazole-resistant strain (MIC=64 µg/mL) was detected in *C. glabrata* by the reference method, but this strain was detected as SDD (MIC=8 µg/mL) with VITEK 2. The same strain was detected as NWT in the case of voriconazole with both the reference method and VITEK 2. Anidulafungin/micafungin resistance was not found with both the reference method and VITEK 2 (Table 2).

While all *C. tropicalis *strains were susceptible to fluconazole with the reference method, resistance was detected in two strains with VITEK 2. Although the sensitivity of voriconazole was 88.9% with both methods, the agreement was low (77.8%) and the minor error rate (16.7%) was high. It was found as a strain of anidulafungin I by both the reference method and VITEK 2. Another strain was found resistant to VITEK 2 (Table 2).

## Discussion

The epidemiology of *Candida* infections is changing; increasing resistance and advances in treatment necessitate widespread use of antifungal susceptibility tests in clinical laboratories, from the point of effective and rapid management of treatment and surveillance monitoring. The reference method standardized by CLSI and EUCAST is difficult in terms of application and interpretation and requires experience, making it difficult to routinely use in clinical laboratories. Therefore, various semi-automatic and automatic commercial antifungal susceptibility testing systems are used to determine antifungal susceptibility. In this study, the antifungal susceptibility of *Candida* strains was determined by the automated VITEK 2 system and the CLSI reference method, and the agreement between the two methods was evaluated.

In the present study, the most successful results were obtained with amphotericin B. Except for one *C. parapsilosis* strain, all of the strains were found to be susceptible to amphotericin B by the reference method, and both essential (93.5%) and categorical agreements (99.3%) were high with the VITEK 2 system. The drug that is the easiest to evaluate in in-vitro antifungal susceptibility tests is amphotericin B. Since the well of the lowest concentration without growth is taken as the MIC, the reading error is low [6]. Although it is the antifungal that was first used and has been in use for a long time, the development of secondary resistance is low and it is thought that this is the reason for obtaining high sensitivity and agreement in this study [20]. Similarly, in another study, essential and categorical agreement with amphotericin B was high [14].

Fluconazole and voriconazole are the azole group drugs used in this study. In particular, fluconazole is important for in vitro susceptibility testing due to its widespread use in the treatment of invasive candidiasis. All *C. albicans* and *C. tropicalis* strains were susceptible to fluconazole using the reference method. However, the major and minor errors observed between the two methods were thought to be related to the higher MIC value of the VITEK 2 system compared to the reference method read at 24 hours [13,15,21]. As seen previously in our center, fluconazole resistance was found to be high in *C. parapsilosis* strains in this study as well [22-25]. It has been shown by microsatellite genotyping that this resistance is nosocomially distributed (Thesis: Semet C; Clonal and clinical relevance in fluconazole-resistant Candida parapsilosis strains isolated from blood cultures. Bursa Uludag University. 2021). Although most of the strains shown to be resistant by the reference method were found to be resistant by VITEK 2, one strain was found susceptible and a major error occurred. Similarly, a *C. glabrata* strain was resistant to fluconazole by the reference method, but not by VITEK 2. Although the VITEK 2 system gives results compatible with reference methods in susceptible strains, data on its performance in resistant strains are insufficient. In a recent study with resistant strains, essential and categorical agreements were suboptimal [26]. Similar to fluconazole, voriconazole resistance was also found to be high in *C. parapsilosis* strains. In addition, the fluconazole-resistant *C. glabrata *strain was found to be resistant to voriconazole as well, and it is considered to be due to cross-resistance between azole drugs [17,21].

In this study, susceptibility of anidulafungin with the CLSI method and micafungin with the VITEK 2 method were examined. Although it is not the same drug, considering that the sensitivity patterns of a drug from echinocandins can be used for all of the members of this group, the two drugs were evaluated only concerning CA [27]. The fact that the CA was high also showed that the use of different drugs in the study did not cause any problems. No resistant strain was detected in both methods and there was no major error. Just as with azoles, the major and minor errors observed between the two methods were related to the higher MIC value produced by the VITEK 2 system than the reference method, which read at 24 hours [28].

## Conclusions

The VITEK 2 system is a fast and highly applicable system, and with these features, it is advantageous for routine laboratories. In this study, the very major error rate was not found to be very high. However, it is problematic that the data on the performance of the VITEK 2 system in rare and resistant strains are insufficient. Although the number of resistant strains found was not much in this study, it is interesting that one fluconazole-resistant strain in *C. parapsilosis* and *C. glabrata* was missed with VITEK 2 and the rates of major and minor errors with fluconazole and voriconazole in *C. parapsilosis* strains are high. There is currently no commercial system that replaces the CLSI or EUCAST reference methods for antifungal susceptibility testing. SensititreYeastOne is the most successful commercial system ever described, as it yields results that are very compatible with the CLSI method. However, it is expensive and difficult to use in centers that have insufficient financial power. The VITEK 2 system, on the other hand, is a system that can be found in many routine laboratories due to its ability to work with bacterial identification and sensitivity. The increase in the data on the performance of the VITEK 2 system in investigating antifungal activity can contribute to the usability of the system for this purpose too. This study presents data that support the literature on the performance of the VITEK 2 system in measuring antifungal activity.

## References

[1] Lass-Flörl C (2009). The changing face of epidemiology of invasive fungal disease in Europe. Mycoses.

[2] Wisplinghoff H, Bischoff T, Tallent SM, Seifert H, Wenzel RP, Edmond MB (2004). Nosocomial bloodstream infections in US hospitals: analysis of 24,179 cases from a prospective nationwide surveillance study. Clin Infect Dis.

[3] Tan TY, Tan AL, Tee NW, Ng LS, Chee CW (2010). The increased role of non-albicans species in candidaemia: results from a 3-year surveillance study. Mycoses.

[4] Bassetti M, Righi E, Costa A (2006). Epidemiological trends in nosocomial candidemia in intensive care. BMC Infect Dis.

[5] Johnson EM (2008). Issues in antifungal susceptibility testing. J Antimicrob Chemother.

[6] (2017). Clinical and Laboratory Standards Institute (CLSI). Reference method for broth dilution antifungal susceptibility testing of yeasts. CLSI guideline M27. Wayne PA: Clinical and Laboratory Standards Institute.

[7] Rodriguez-Tudela JL, Arendrup MC, Barchiesi F (2008). EUCAST definitive document EDef 7.1: method for the determination of broth dilution MICs of antifungal agents for fermentative yeasts. Clin Microbiol Infect.

[8] Morace G, Polonelli L (2005). Voriconazole activity against clinical yeast isolates: a multicentre Italian study. Int J Antimicrob Agents.

[9] Cuenca-Estrella M, Gomez-Lopez A, Mellado E, Rodriguez-Tudela JL (2005). Correlation between the procedure for antifungal susceptibility testing for Candida spp. of the European Committee on Antibiotic Susceptibility Testing (EUCAST) and four commercial techniques. Clin Microbiol Infect.

[10] Clinical and Laboratory Standards Institute (CLSI (2018). Clinical and Laboratory Standards Institute (CLSI). Method for antifungal disk diffusion susceptibility testing of yeasts. 3rd ed. CLSI guideline. M44. Wayne PA: Clinical and Laboratory Standards Institute.

[11] Hadrich I, Ayadi A (2018). Epidemiology of antifungal susceptibility: review of literature. J Mycol Med.

[12] Bourgeois N, Dehandschoewercker L, Bertout S, Bousquet PJ, Rispail P, Lachaud L (2010). Antifungal susceptibility of 205 Candida spp. isolated primarily during invasive candidiasis and comparison of the Vitek 2 system with the CLSI broth microdilution and Etest methods. J Clin Microbiol.

[13] Pfaller MA, Diekema DJ, Procop GW, Rinaldi MG (2007). Multicenter comparison of the VITEK 2 yeast susceptibility test with the CLSI broth microdilution reference method for testing fluconazole against Candida spp. J Clin Microbiol.

[14] Pfaller MA, Diekema DJ, Procop GW, Rinaldi MG (2007). Multicenter comparison of the VITEK 2 antifungal susceptibility test with the CLSI broth microdilution reference method for testing amphotericin B, flucytosine, and voriconazole against Candida spp. J Clin Microbiol.

[15] Posteraro B, Martucci R, La Sorda M (2009). Reliability of the Vitek 2 yeast susceptibility test for detection of in vitro resistance to fluconazole and voriconazole in clinical isolates of Candida albicans and Candida glabrata. J Clin Microbiol.

[16] VITEK® 2 AST Cards (2021). VITEK® 2 AST cards. Cards for antimicrobial susceptibility testing. https://www.biomerieux-diagnostics.com/vitekr-2-ast-cards-0.

[17] Cilo BD, Ağca H, Ener B (2019). Identification of Candida parapsilosis complex strains isolated from blood samples at species level and determination of their antifungal susceptibilities [Article in Turkish]. Turk Mikrobiyol Cem Derg.

[18] (2020). CLSI. Performance standards for antifungal susceptibility testing of yeasts. CLSI supplement M60. Wayne, PA: Clinical and Laboratory Standards Institute.

[19] (2020). CLSI. Epidemiological cutoff values for antifungal susceptibility testing. CLSI supplement M59. Clinical and Laboratory Standards Institute.

[20] Vincent BM, Lancaster AK, Scherz-Shouval R, Whitesell L, Lindquist S (2013). Fitness trade-offs restrict the evolution of resistance to amphotericin B. PLoS Biol.

[21] Pfaller MA, Diekema DJ, Procop GW, Rinaldi MG (2013). Comparison of the Vitek 2 yeast susceptibility system with CLSI microdilution for antifungal susceptibility testing of fluconazole and voriconazole against Candida spp., using new clinical breakpoints and epidemiological cutoff values. Diagn Microbiol Infect Dis.

[22] Gürcüoğlu E, Ener B, Akalin H (2010). Epidemiology of nosocomial candidaemia in a university hospital: a 12-year study. Epidemiol Infect.

[23] Kazak E, Akın H, Ener B (2014). An investigation of Candida species isolated from blood cultures during 17 years in a university hospital. Mycoses.

[24] Dalyan Cilo B, Topaç T, Ağca H, Sağlam S, Efe K, Ener B (2018). Comparison of Clinical Laboratory Standards Institute (CLSI) and European Committee on Antimicrobial Susceptibility Testing (EUCAST) broth microdilution methods for determining the susceptibilities of Candida isolates [Article in Turkish]. Mikrobiyol Bul.

[25] Arikan-Akdagli S, Gülmez D, Doğan Ö (2019). First multicentre report of in vitro resistance rates in candidaemia isolates in Turkey. J Glob Antimicrob Resist.

[26] Wong KY, Gardam D, Boan P (2019). Comparison of Vitek 2 YS08 with Sensititre YeastOne for Candida susceptibility testing. Pathology.

[27] Berkow EL, Lockhart SR, Ostrosky-Zeichner L (2020). Antifungal susceptibility testing: current approaches. Clin Microbiol Rev.

[28] Peterson JF, Pfaller MA, Diekema DJ, Rinaldi MG, Riebe KM, Ledeboer NA (2011). Multicenter comparison of the Vitek 2 antifungal susceptibility test with the CLSI broth microdilution reference method for testing caspofungin, micafungin, and posaconazole against Candida spp. J Clin Microbiol.

